# **Impact of neoadjuvant cetuximab on liver function and tumor reduction in a murine model of selective portal vein ligation**

**DOI:** 10.1007/s10585-025-10386-7

**Published:** 2025-12-27

**Authors:** Guillermo Tirado-Rodríguez, Inmaculada Ruiz-Montesinos, Sira Iturrizaga, Amador García Ruiz de Gordejuela, Paula Aizpiolea, Daniel Alonso-Alconada, Ignacio García-Alonso, Borja Herrero de la Parte

**Affiliations:** 1 Service of Urology, Lozano Blesa University Clinical Hospital, Zaragoza, Spain; 2 Department of Surgery and Radiology and Physical Medicine, University of the Basque Country UPV/EHU, Bilbao, Spain; 3 Service of General and Digestive Surgery, Donostia University Hospital, Donostia, Spain; 4 Service of Clinical Analysis, Galdakao-Usansolo Hospital, Galdakao, Spain; 5 Service of General and Digestive Surgery, Miguel Servet University Hospital, Zaragoza, Spain; 6 Department of Cell Biology and Histology, University of the Basque Country UPV/EHU, Bilbao, Spain; 7 Biobizkaia Health Research Institute, Barakaldo, Spain

**Keywords:** Cetuximab, Liver regeneration, Colorectal cancer liver metastasis, Neoadjuvant therapy, Portal vein ligation, Animal study

## Abstract

Colorectal cancer mortality is largely driven by metastatic spread, with colorectal liver metastases (CRLM) being the most frequent. Treatment often combines surgery and systemic therapy, including anti-EGFR agents such as cetuximab. This study evaluated the cytoreductive effect of cetuximab on CRLM and its influence on liver function and regeneration in a selective portal vein ligation (PVL) model. Sixty-six male WAG/RijHsd rats were used. Seven days before PVL, twelve tumor-bearing animals received intra-arterial cetuximab, while other six received vehicle (saline). Additional groups included tumor-bearing rats without PVL, rats undergoing PVL alone, and untreated controls (n = 6 each). Seven days after PVL, the future remnant liver volume (%FRL) and hepatocyte replication were quantified. Serum markers for hepatic, renal, and systemic injury were analyzed, and tumor volume was assessed by ultrasound before and after PVL. The regenerative response following PVL was not significantly affected by cetuximab, with %FRL reaching 80–90%. However, hepatocyte nuclei exhibited a smaller mean area compared with non-treated animals (45.35 ± 9.09 vs. 43.54 ± 9.87 μm²; *p* < 0.001). Liver function remained preserved, although glucose levels decreased after PVL. Cetuximab significantly reduced tumor growth driven by hepatic regeneration (1.12 ± 0.45 vs. 0.53 ± 0.16 mL; *p* < 0.01). Neoadjuvant intra-arterial cetuximab does not impair liver regeneration after PVL while effectively limiting tumor proliferation linked to regenerative stimuli. These findings support its perioperative safety and potential use to prevent tumor recurrence in patients with CRLM undergoing staged hepatectomy.

## Introduction

Colorectal cancer (CRC) is one of the most common malignancies worldwide and a leading cause of cancer-related death. The liver is the most frequent site of metastases, occurring in approximately 50–60% of CRC patients during the disease course. In patients with colorectal liver metastases (CRLM), surgical resection remains the gold standard for cure, achieving 40–60% 5-year survival rates in selected cases. However, only 15–25% of patients are suitable for resection at diagnosis, and recurrence—often hepatic—remains a major challenge [1–3]. When resection is not initially feasible, different strategies aimed to render patients operable while preserving adequate liver function are available. An adequate future remnant liver (FRL) is critical to avoid postoperative liver failure (PLF), a potentially fatal complication. In healthy livers, resection of up to 75–80% may be tolerated [4–7]; however, prior chemotherapy reduces regenerative capacity, lowering safe thresholds to 40–50% of FRL [4, 7–9].

Various techniques have been developed to induce hypertrophy before surgery, including portal vein embolization (PVE), selective portal vein ligation (PVL), and associating liver partition and portal vein ligation for staged hepatectomy (ALPPS) [10]. All of them are particularly relevant in patients with bilobar metastases suitable for a two-stage approach: ligation/embolization of the portal vein in the most affected lobe combined with clearance of lesions in the contralateral lobe. After 3–6 weeks, once hypertrophy of the FRL occurs, the second stage removes the remaining tumor-bearing liver [10, 11]. Although these techniques improve resectability, there is growing concern that rapid liver regeneration may promote tumor progression [12, 13], since hepatic hypertrophy induces a proliferative microenvironment rich in growth factors, cytokines, and proangiogenic signals, which may stimulate dormant micrometastases or residual tumor cells, accelerating recurrence. In rodent models, liver regeneration after hepatectomy or PVL has been associated with increased growth of occult metastases, mediated in part by vascular endothelial growth factor (VEGF) and epidermal growth factor receptor (EGFR) signaling pathways [14–16].

EGFR is overexpressed in many CRCs, particularly those without RAS mutations, and plays a key role in tumor cell proliferation, survival, and migration [17–19]. Cetuximab, a chimeric monoclonal antibody targeting EGFR, has been approved for RAS wild-type metastatic CRC in combination with chemotherapy or as monotherapy in selected cases [20]. Other monoclonal agents used for metastatic CRC include aflibercept and ramucirumab, which also target VEGF or its receptors, and panitumumab (human EGFR monoclonal antibody) [21]. Recently, fruquintinib (selective inhibitor of EGFR tyrosine kinase) and trifluridine-tipiracil (trifluridine is a DNA-damaging agent, tipiracil delays trifluridine metabolization) in combination with bevacizumab have also been approved [22, 23].

While systemic chemotherapy is often used to control micrometastatic disease, its hepatotoxic effects may compromise liver regeneration. Targeted agents like cetuximab, with a different toxicity profile [24, 25] do not affect healthy liver tissue. Therefore, they could offer an alternative strategy to prevent the activation of latent metastases without impairing hypertrophy, unlike bevacizumab [26]. However, the optimal timing, combination with other therapies, and impact on long-term oncologic outcomes remain uncertain. This concept is particularly relevant in the perioperative management of CRLM patients undergoing PVL, PVE, or ALPPS.

Therefore, strategies integrating liver regeneration techniques with targeted biological therapy warrant further investigation. This study explores, in a rat model of selective PVL and FLR hypertrophy, the hypothesis that perioperative cetuximab may mitigate tumor progression associated with hepatic regeneration, with potential translational relevance for patients with CRCLM.

## Methods

Sixty-six male albino rats (WAG/RijHsd strain), 3–4 months old and weighing 255.3 ± 3.87 g, were housed under controlled temperature and humidity, with a 12-hour light/dark cycle, and given food and water ad libitum. The number of animals per group was calculated using GRANMO sample size calculator v8.00 (online free access), based on expected effect size and variability from preliminary data. The minimum number of animals required to achieve statistical significance was used, in accordance with the principles of the 3Rs (Replacement, Reduction, and Refinement). Five groups were established: control (*n* = 6), healthy animals without intervention; tumor (*n* = 6), tumor-bearing animals without intervention; PVL (*n* = 6), healthy animals subjected only to portal vein ligation; cetuximab (*n* = 12), tumor-bearing animals undergoing PVL and receiving intra-arterial cetuximab prior to PVL; and vehicle (*n* = 12), tumor-bearing animals undergoing PVL and receiving the same intra-arterial volume of saline. For tumor induction, animals were anaesthetized with 1.5% isoflurane, and 0.05 ml of a cell suspension containing 500,000 cells/ml was administered to both the left lateral lobe (LLL) and the right paramedian lobe (RPML) [27]. Twenty-one days after tumor induction, the animals underwent abdominal ultrasonography to verify the correct development and implantation of CRLM; then, the animals were randomly assigned to the different experimental groups.

**Fig. 1:**
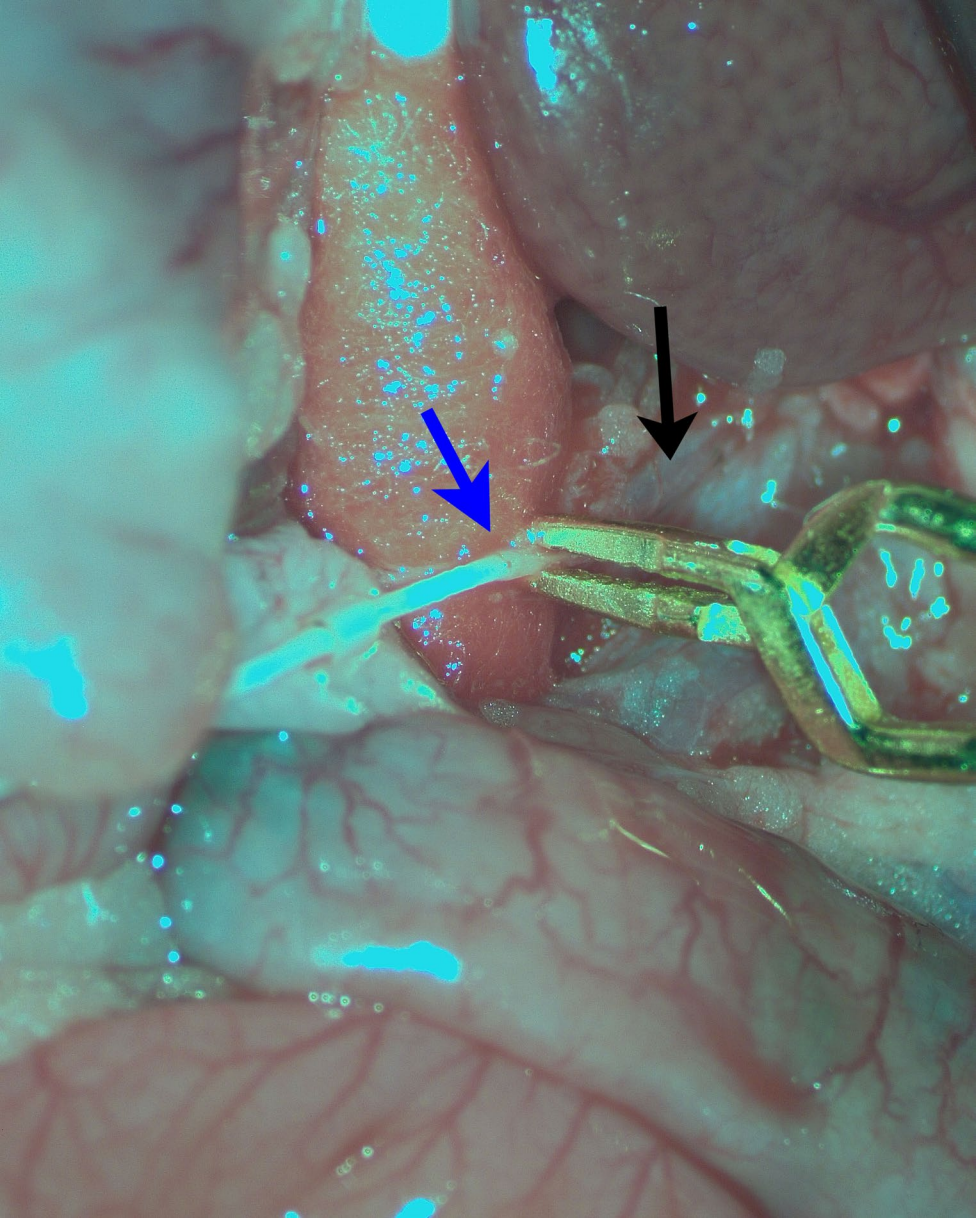
Intra-arterial infusion of cetuximab. Note the microcatheter introduced into the splenic artery (blue arrow) and secured to the splenic artery by a Yasargil vascular clip; black arrow shows de celiac trunk

In the neoadjuvant and vehicle groups, treatment was selectively delivered via a catheter inserted through the splenic artery into the hepatic artery [28]. Briefly, the splenic artery was punctured with a 30G needle, and a perfluorocarbon catheter (0.4 mm outer, 0.2 mm inner diameter; ref. 72-9030, Harvard Apparatus, USA) was advanced into the common hepatic artery. To prevent extravasation, the catheter was secured with a Yasargil clip (shown in Fig. 1). Then, saline or cetuximab were slowly infused (2–3 min). After removal, the splenic artery was compressed for 5 min to achieve hemostasis.

Cetuximab (Erbitux^®^, Merck, Darmstadt, Germany) was administered at a dose of 114 mg/m^2^ [29]. To calculate the animal’s body area the “Meeh’s” formula was used: A = K x W2/3; where A, represents the body area; K, is an animal-specific constant (in the case of rats it is 9.83); and W, is the individual weight of the animal [30]. Seven days after cetuximab or saline administration, PVL was performed. For this purpose, we used a PVL model previously described by our group and other researchers, depriving the portal venous supply to LLL and left paramedian lobe (LPML) which amounts to approximately 40–50% of the total liver tissue [31, 32]. Under 1.5% isoflurane anesthesia, a 25–30 mm median laparotomy was performed to expose the posterior aspect of the liver. Under a surgical microscope, the left portal branch was identified, isolated and ligated with 6/0 silk. After verifying the absence of hemorrhage and the change of color in the liver affected by PVL, the laparotomy was closed by a continuous suture with 4/0 polyglycolic acid absorbable suture material (Coated VICRYL^®^, ref.: J310H Ethicon Inc., USA) for the muscular layer and 3/0 silk (ref.: 55346, Lorca-Marín S.A., Spain) for the skin. Following each experimental procedure, 2 mg/kg meloxicam (Loxicom^®^, Noorbrok; Newry, UK) was administered subcutaneously and the animals remained under a heat source until full recovery.

Seven days after performing the portal vein ligation (PVL), tumor volume was assessed by ultrasound, and blood and liver tissue samples were collected under 1.5% isoflurane anesthesia. An 18 MHz ultrasound probe was used to locate CRLM, and axial and sagittal lengths of each tumor implant were measured to calculate tumor volume.

Afterwards, using a 21G needle, the total circulating blood volume was withdrawn from the abdominal aorta. The blood was then centrifuged for 10 min at 3000 rpm in SST™ II Advance tubes (BD Vacutainer^®^, New Jersey, USA). The serum was aliquoted and stored at -80 °C until analysis, which was carried out using a Cobas^®^ 8000 c702 analyzer (Roche Diagnostics GMBH, Mannheim, Germany). The following biochemical markers were measured to further assess the hepatic function and metabolic status: alanine aminotransferase (ALT), aspartate aminotransferase (AST), alkaline phosphatase (ALP), glucose, total bilirubin (TBil), albumin, cholinesterase, cholesterol, and total protein, all from Roche Diagnostics GMBH.

Hepatic and tumor tissues, depending on the group, were collected immediately after blood collection. The entire liver was removed and separated into atrophic tissue (from PVL sites: LLL and LPML) and hypertrophied tissue (RPML, right lateral lobe (RLL), and caudate lobe (CL)). Both types of fresh liver tissue were weighed separately, and the percentage of future remnant liver (%FRL) was calculated using the formula: %FRL = (hypertrophic liver weight / total liver weight) × 100. The liver parenchyma was then preserved in 4% formaldehyde and embedded in paraffin. Histological Sections (5 μm) were stained with hematoxylin-eosin. Hepatocyte nuclear area was measured to assess hepatic regeneration. This measurement was done on at least 120 hepatocyte nuclei per animal, using Leica Application Suite (LAS) software (Leica Microsystems, Wetzlar, Hesse, Germany), with samples blinded to the researcher performing the analysis.

Data collected throughout the experimental process were initially recorded on a data sheet and then analyzed using Prism^®^ software, version 10.1.1 (GraphPad Software, San Diego, California, USA). Data were checked for normality and are presented as mean and standard deviation. To compare tumor volumes, a t-test was used. For other analyses involving three or more experimental groups, data were subjected to analysis of variance (ANOVA); if significant differences were found, Tukey’s multiple comparison test was applied. A significant level of 95% (*p* < 0.05) was used for all statistical tests.

## Results

### Liver regeneration: %FRL and hepatocyte nuclear area

All procedures were well tolerated, with no signs indicating alterations in animal welfare. The animals did not exhibit signs of abdominal bleeding, peritoneal ascites, or significant weight loss.

**Fig. 2:**
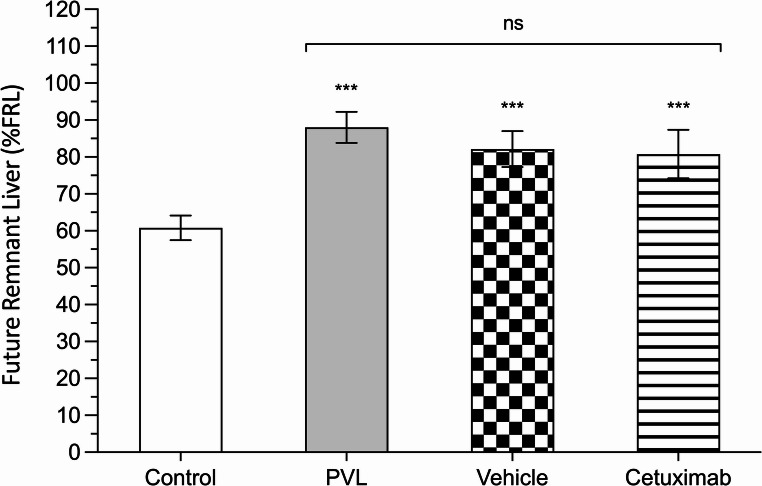
Percentage of Future Remnant Liver (%FRL). Values are shown for control rats (control; white bar), rats subjected to selective portal vein ligation (PVL; gray bar), rats treated with saline (vehicle; checked bar), and rats treated with cetuximab (cetuximab; striped bar) (***: *p* < 0.001; n.s.: *p* > 0.05)

PVL significantly increased %FRL as may be seen in every group including this surgical procedure (*p* < 0.001, Fig. 2). These finding parallels what is seen when assessing liver volume in groups including PVL: hepatic volume was increased by almost 30% in PVL group (88.02 ± 4.19%) when compared to the control group (60.79 ± 3.23%). The effect of PVL on liver growth was not affected by intraarterial infusion of either vehicle (82.16 ± 4.86%) or cetuximab (80.73 ± 6.58&).

The groups receiving intra-arterial fluid infusion (vehicle or cetuximab) did not show significant differences in %FRL compared to the PVL-only group (82.16 ± 4.86%, 80.73 ± 6.58%, and 88.02 ± 4.19%, respectively; *p* > 0.05).

**Fig. 3:**
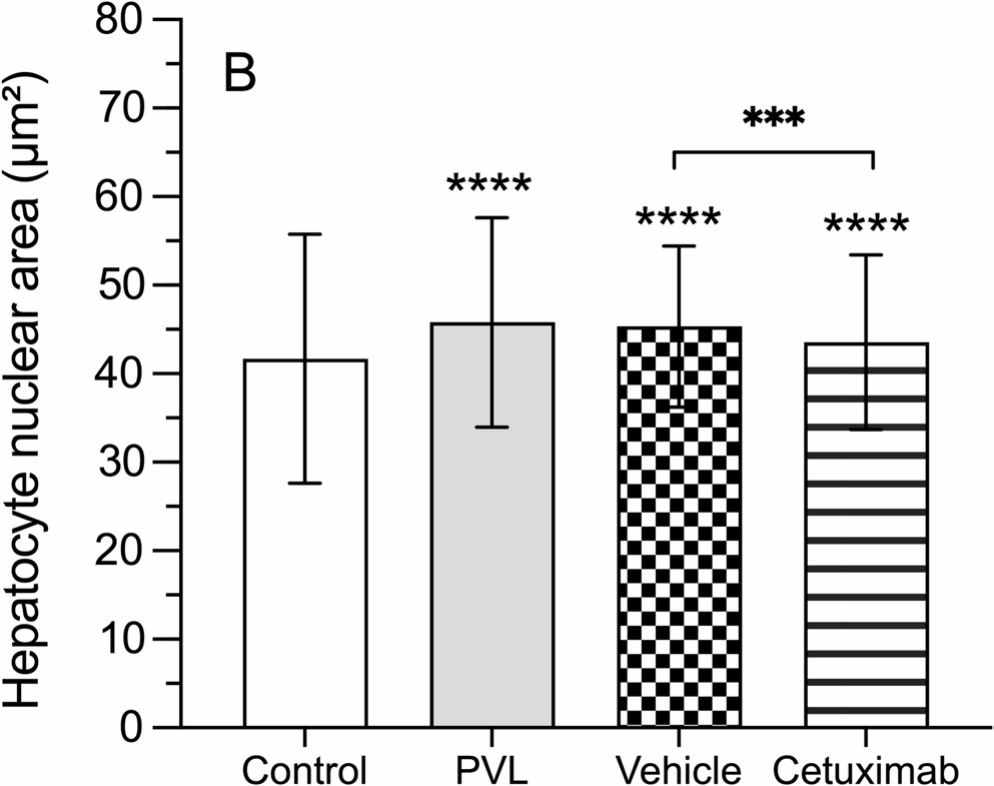
Analysis of hepatocyte proliferation. Representative histological section of the hypertrophic liver lobe stained with hematoxylin-eosin (**A**), showing hepatocyte nuclei highlighted in yellow during nuclear area assessment; the scale bar represents 50 μm. Graphical representation of hepatocyte nuclear area (µm²) (**B**), quantified in the paramedian lobe (hypertrophic) 7 days after selective portal vein ligation (PVL; gray bar). Asterisks above the bars indicate statistically significant differences compared to the control group, while asterisks above the top box indicate statistically significant differences between the vehicle group (checked bar) and the cetuximab group (horizontal striped bar) (***: *p* < 0.001; ****: *p* < 0.0001)

In control animals the mean nuclear area of the hepatocytes was 41.69 ± 14.05 μm². Following PVL this parameter was significantly increased (*p* < 0.0001) (Fig. 3). Tumor-bearing animals that received intra-arterial vehicle infusion did not show significant changes in hepatocyte’s nuclear area compared to the PVL-only group (45.35 ± 9.09 vs. 45.8 ± 11.81 μm²; *p* > 0.05). However, cetuximab reduced the increase in nuclear size induced by PVL (43.54 ± 9.87 μm²; *p* < 0.001), though it remained over control values (*p* < 0.05).

**Fig. 4:**
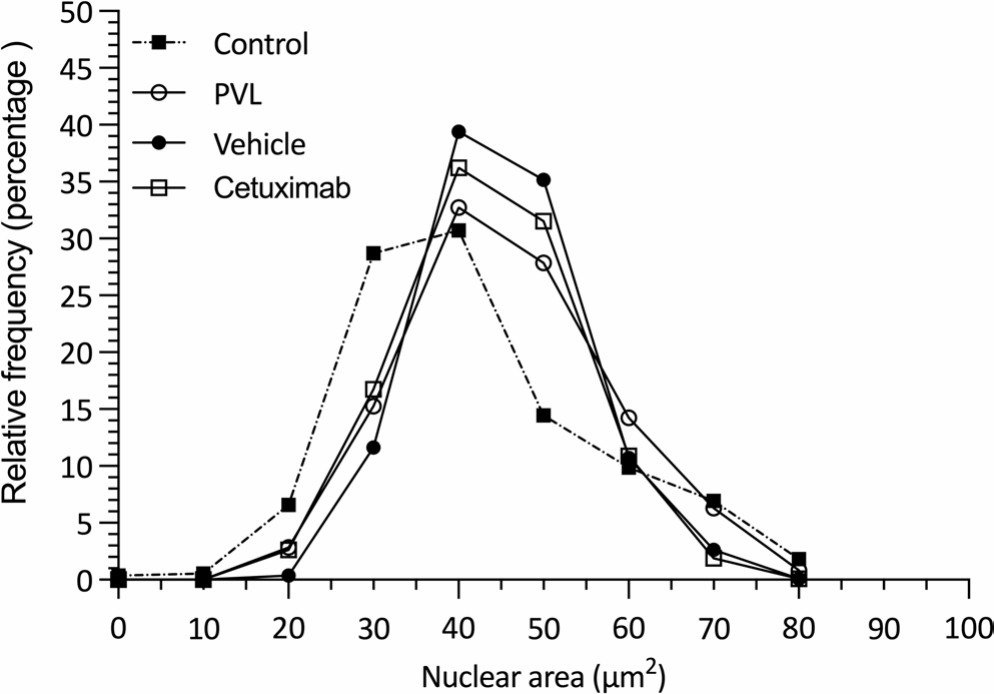
Histogram of relative frequencies of hepatocyte nuclear area. Nuclear area (µm²) quantified in hepatocytes from the paramedian lobe (hypertrophic), 7 days after selective portal vein ligation (PVL): control group (dotted line and black square), PVL group (solid line and white circle), vehicle group (solid line and black circle), and cetuximab group (solid line and white square)

The values of hepatocyte’s nuclear area have also been distributed in a relative frequency histogram which is a common tool to visualize hepatocyte regeneration (Fig. 4). The three groups subjected to PVL show a right shift in the curve if compared to control animals (*p* < 0.0001), which is consistent with liver regeneration. Intra-arterial administration of vehicle or cetuximab does not modify the pattern (area under the curve *p* > 0.05).

### Hepatic function and metabolic status

**Fig. 5:**
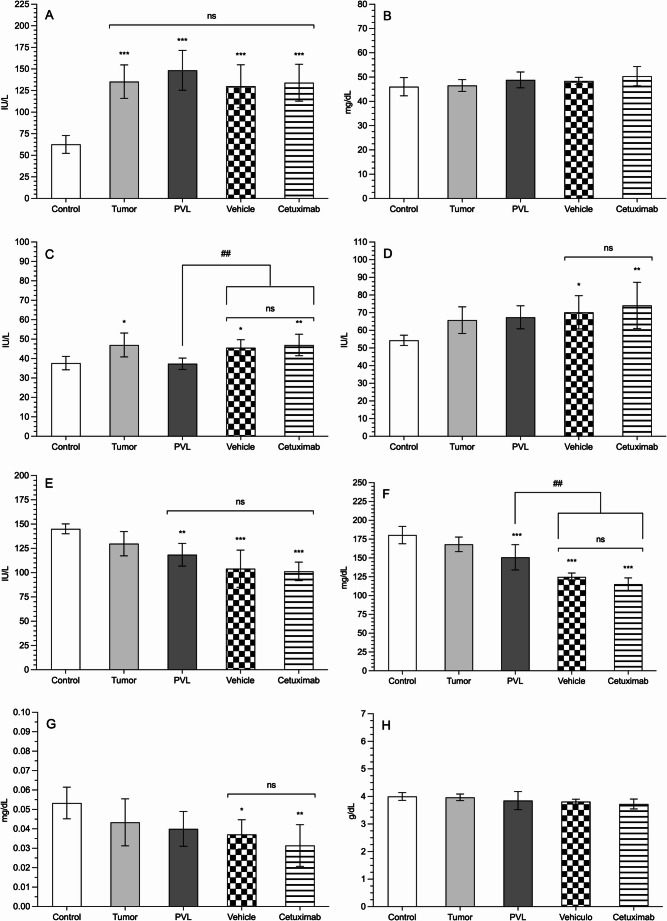
Serum levels of cholinesterase (**A**), cholesterol (**B**), alanine aminotransferase (ALT; **C**), aspartate aminotransferase (AST; **D**), alkaline phosphatase (ALP; **E**), glucose (**F**), total bilirubin (TBil; **G**), and albumin (**H**). Units are expressed as international units per liter (IU/L) for ALT, AST, and ALP; milligrams per deciliter (mg/dL) for glucose and TBil; and grams per deciliter (g/dL) for albumin. Asterisks indicate statistically significant differences compared with the control group (*p < 0.05, **p < 0.001, ***p < 0.001), while hash marks indicate statistically significant differences between groups as denoted by the bar (##*p* < 0.01); ns: not significant (*p* > 0.05)

Of the eight liver markers analyzed (Table 1), cholinesterase has been the most altered (Fig. 5A). Both the presence of tumor in the liver and PVL have doubled the values of control animals (135 ± 19, 149 ± 23 vs. 63 ± 10 IU/L; *p* < 0.001). Neither the vehicle nor cetuximab have significantly modified this increase (130 ± 25, 134 ± 21 IU/L). A similar pattern is seen in total protein values (Fig. 5B).

**Table 1:** Biochemical analysis values in the experimental groups. Units are expressed as international units per liter (IU/L) for Alanine aminotransferase (ALT), aspartate aminotransferase (AST), alkaline phosphatase (ALP), and cholinesterase; in milligrams per deciliter (mg/dL) for glucose, cholesterol, and total bilirubin (TBil); and in grams per deciliter (g/dL) for total protein and albumin. Data are presented as mean and standard deviation

	Control	Tumor	PVL	Vehicle	Cetuximab
Cholinesterase (IU/L)	63 ± 10	135 ± 19	149 ± 23	130 ± 25	134 ± 21
Cholesterol (mg/dL)	46 ± 3.8	47 ± 2.4	49 ± 3.3	48 ± 1.5	50 ± 4
ALT (IU/L)	38 ± 3.4	47 ± 6.1	37 ± 2.9	46 ± 4.1	47 ± 5.5
AST (IU/L)	54 ± 2.9	66 ± 7.6	67 ± 6.5	70 ± 9.4	74 ± 13
ALP (IU/L)	145 ± 5.1	130 ± 13	119 ± 12	104 ± 19	101 ± 9.6
Glucose (mg/dL)	180 ± 12	168 ± 9.6	151 ± 17	125 ± 5.1	115 ± 8.4
TBil (mg/dL)	0.053 ± 0.008	0.043 ± 0.012	0.04 ± 0.009	0.037 ± 0.007	0.031 ± 0.01
Albumin (g/dL)	4 ± 0.14	4 ± 0.12	3.9 ± 0.33	3.8 ± 0.09	3.7 ± 0.18
Total protein (g/dL)	4.9 ± 0.19	5.3 ± 0.29	5.3 ± 0.15	5.3 ± 0.17	5.3 ± 0.18

Both ALT and AST were increased in tumor bearing animals, while PVL did not affect these enzymes (Fig. 5C–D). However, when the hepatic artery was cannulated a significant (though not clinically relevant) increase was observed. Cetuximab did not show any effect.

ALP (Fig. 5E) was not affected by tumor development. However, animals subjected to PVL showed a significant decrease in the enzyme (*p* < 0.01), been slightly greater when arterial infusion was performed (*p* < 0.001).

Glucose levels (Fig. 5F) showed a pattern like ALP, but the further decrease in vehicle (125 ± 5.1 mg/dL) and cetuximab (115 ± 8.4 mg/dL) groups here reached statistical significance (PVL 151 ± 17 mg/dL; *p* < 0.01).

TBil levels (Fig. 5G) were close to control values (0.053 ± 0.008 mg/dL) in the tumor and PVL groups (*p* > 0.05). However, they were slightly lower in the groups with higher surgical stress (vehicle and cetuximab), with values dropping below 0.04 mg/dL (0.037 ± 0.007 and 0.031 ± 0.01 mg/dL, respectively; *p* < 0.05).

Finally, serum albumin and cholesterol remained stable with no significant differences among any of the groups.

### Tumor growth

**Fig. 6:**
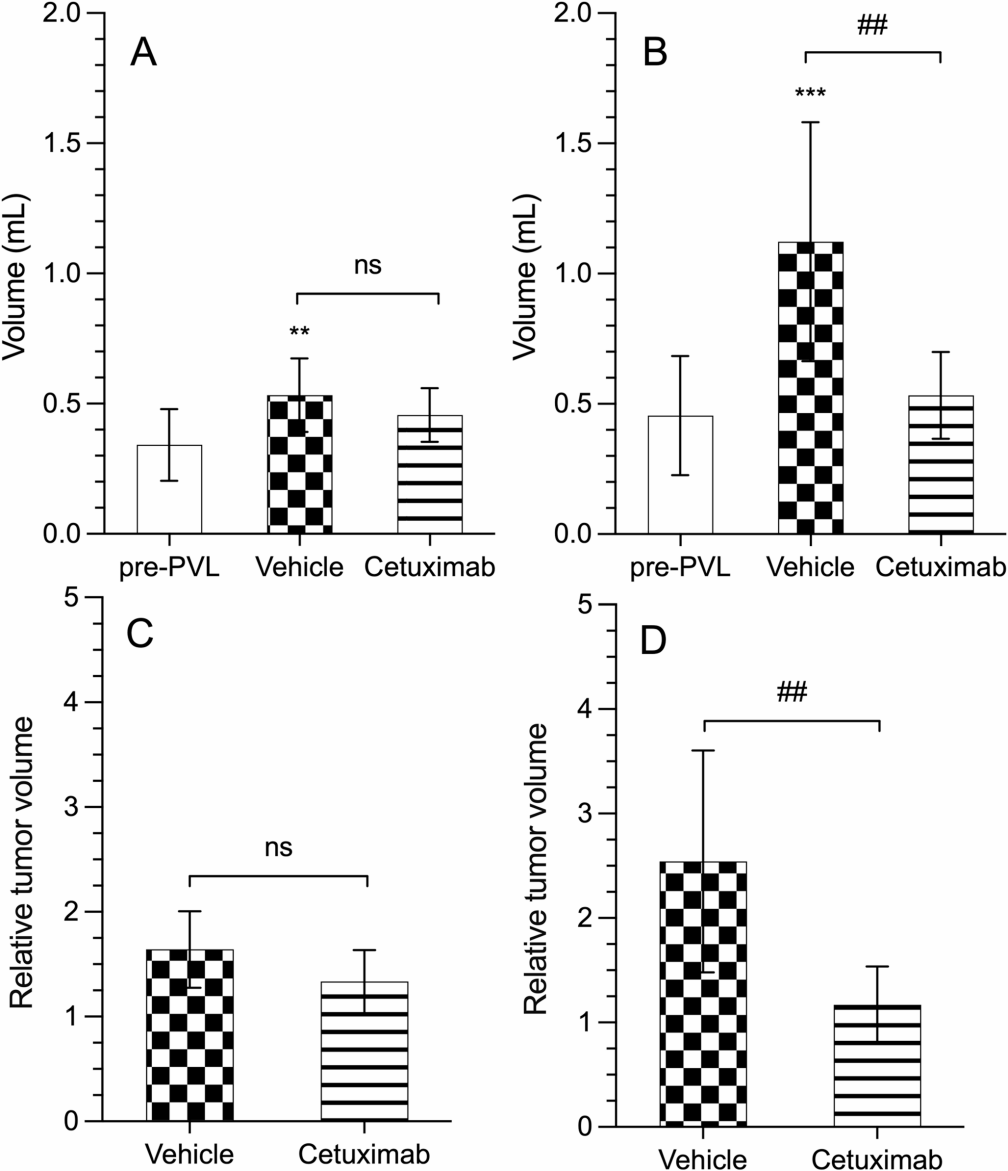
Tumor volume (mL) of implants located in the left lateral lobe (LLL; **A**) and the right paramedian lobe (RPML; **B**), as well as relative tumor growth in the LLL (**C**) and RPML (**D**). Tumor assessment was performed by ultrasound before selective portal vein ligation (PVL) (pre-PVL; white bar) and after PVL in animals treated with saline (vehicle; checkered bar) or cetuximab (striped bar). Asterisks indicate statistically significant differences compared with pre-PVL volume (**p < 0.01, *p < 0.001), while hash marks indicate statistically significant differences between groups as denoted by the bar (##*p* < 0.01). ns: not significant (*p* > 0.05)

Regarding the assessment of tumor volume, we observed that the dynamic behavior of tumor volume differed between the two tumor implants depending on the hepatic lobe in which they were implanted, the LLL (atrophic lobe) or the RPML (hypertrophic lobe) (shown in Fig. 6A–C and B–D, respectively).

No differences were observed in the LLL-tumor volume when comparing the vehicle and cetuximab groups following PVL (0.53 ± 0.14 vs. 0.45 ± 0.1 ml, respectively) (shown in Fig. 6A). In the vehicle group, a slight increase in tumor volume was observed compared with the size prior to PVL. Similarly, no differences were found when comparing the relative increase in tumor volume (*p* > 0.05) (shown in Fig. 6C).

Concerning the RPML, the difference in tumor size between the vehicle-treated and cetuximab-treated groups was highly significant, since the vehicle-treated group had twice the tumor volume of the cetuximab-treated group (1.12 ± 0.45 vs. 0.53 ± 0.16 ml; *p* < 0.01) (shown in Fig. 6B). The tumor volume in the vehicle group was approximately 2.5 times larger than pre-PVL tumor volume. In contrast, the cetuximab group showed only a 1.17-fold increase in tumor volume (shown in Fig. 6D).

## Discussion

The use of monoclonal drugs and their impact on liver regeneration is an area of interest in oncology, particularly in the treatment of patients with CRLM. Monoclonal anti-EGFR antibodies, such as cetuximab or panitumumab, have not shown issues related to wound healing, specific liver toxicity, or other adverse effects that could compromise the safety of oncological surgical interventions [33, 34]. This aspect is crucial in scenarios where the percentage of liver remnant is insufficient. In the present study, no adverse events related to the chemotherapeutic agent were observed, and the animals tolerated the treatment satisfactorily.

Their therapeutic indication allows for their use both as adjuvant and neoadjuvant therapy, the latter requiring discontinuation 3–4 weeks before the tumor surgery [35]. In a neoadjuvant setting, prior to surgical liver resection, EGFR inhibitors are supposed to convert unresectable CRLM into resectable cases, improving response rates and increasing the number of patients eligible for oncological surgery [36].

However, recent clinical evidence, such as the long-term results from the New EPOC trial, has challenged the benefit of perioperative anti-EGFR therapy in resectable CRLM, showing worse progression-free and overall survival when cetuximab was added to standard chemotherapy [37]. These findings highlight the complex interaction between EGFR blockade and liver regeneration, underscoring the need for mechanistic models capable of isolating these effects. In this context, our rat PVL model provides translational insight into how hepatic hypertrophy may modulate tumor behavior under EGFR inhibition, helping to interpret why perioperative cetuximab could yield unexpected oncological outcomes in clinical practice. In our experimental model, PVL is performed 1 week after cetuximab infusion, as the animals used exhibit faster healing rates compared to humans [38, 39]. This timing was supported by previously studies published by our group as well as many other authors [31, 32, 40–42]; liver regeneration in rats is completed after seven days, with no further increases in liver volume.

The intra-arterial route was selected to achieve higher local drug concentrations in the liver while minimizing systemic exposure, thus maximizing the antitumor effect in the hepatic tissue. The dose was determined based on previous experimental studies demonstrating both safety and efficacy in achieving EGFR blockade without inducing hepatotoxicity [29].

It is clearly established that cetuximab is effective when treating CRLM well-defined and categorized through imaging tests. But considering the critical role of EGFR in tumor cell proliferation and survival, EGFR inhibitors may help prevent tumor recurrence or even inhibit the proliferation of undiagnosed micrometastases or the activation of dormant metastases. These latter scenarios are the most concerning when discussing oncological surgery and liver regeneration, as the initial surgical act involves resection of hepatic parenchyma and CRLM [43].

After hepatectomy, the balance between hepatic damage following surgery and liver repair by hepatocyte regeneration ensures survival. The activation of fibroblast growth factor 2 (FGF-2) and VEGF is essential for liver regeneration. In this model, a 70% resection is used according to Bönninghoff et al. as it achieves an optimal peak in VEGF production [44].

Regarding the role of cetuximab in tumor development, a clear antitumoral effect was observed. The mean size of metastases in animals treated with cetuximab was reduced in both hepatic lobes compared with the vehicle. However, this reduction reached statistical significance only in the implant located in the LMD and not in the atrophic lobe. Overall, tumor growth was 46% lower in the cetuximab group, indicating that hypertrophy of the LMD did not impair the antitumoral efficacy of the drug. Although tumors still increased in size compared with their pre-PVL measurements—indicating that cetuximab did not eradicate metastatic lesions in this model—this finding should be interpreted considering several biological and methodological considerations. Complete radiological or pathological responses to cetuximab have indeed been reported across tumor types [45, 46], including CRLM, but such outcomes typically arise from multidrug regimens such as FOLFOX or FOLFIRI combined with cetuximab, administered over multiple cycles. In contrast, our model relied on a single intra-arterial dose of cetuximab given as monotherapy. Moreover, PVL induces profound hepatic hypertrophy accompanied by the release of trophic and regenerative factors, which—while essential for liver regeneration—simultaneously exert potent pro-tumorigenic stimuli. Therefore, despite only achieving a partial response, the approximately 50% reduction in tumor progression highlights the substantial antitumoral capacity of cetuximab, even under strong proliferative pressure, and importantly, without compromising liver regeneration.

In this study, analysis of the remaining liver volume shows that cetuximab does not significantly affect %FRL, so this drug can be considered safe regarding hepatic mass recovery post-PVL in the present experimental model; these finding correlates to results published by Inoue et al. [47].

The hepatocyte volume in the liver, both in humans and mice, approximately doubles with the duplication of DNA content [48–50]. In pathological conditions, liver polyploidization is directly associated with the extent of damage, so determining nuclear area provides an indirect measure of the amount of DNA in the nucleus [50–53]. In this model, cetuximab negatively affected this parameter; the nuclear size quantified in animals treated with cetuximab was significantly smaller than in the vehicle group, which is expected given cetuximab’s role as a chimeric anti-EGFR monoclonal antibody.

Considering the results of hepatocyte nuclear area and %FRL, it may look as if both results are contradictory. However, this discrepancy can be explained by the fact that early phases of hepatic regeneration depend on hepatocyte hypertrophy, and despite the ploidy of liver cells, the number of nuclei decreases according to the model of Miyaoka et al. [54].

To mitigate this potential deleterious effect on DNA synthesis and, consequently, on cell proliferation, therapies that stimulate these processes in conjunction with the proliferative stimulus resulting from PVL could be employed. As Gutiérrez Sáenz de Santamaría et al. observed that folate promotes a greater increase in %FRL 7 days after PVL [32], it could be interesting to check if combining folic acid with cetuximab achieves better and/or faster hepatic regeneration compensation.

Regarding hepatic function, enzymes of cytolysis (AST and ALT) and cholestasis (ALP) did not show significant variations when cetuximab was introduced as an experimental variable, indicating that its administration with the purpose of inhibiting tumor tissue did not cause additional damage to hepatic tissue. ALT values showed that the enzyme increase, and therefore the rise in hepatic cytolysis, is more strongly dependent on the presence of tumor implants, as reported by Zhang et al. [55]. Meanwhile, ALP tends to decrease compared to the control, and although it might seem that it should increase due to the tumor, cholestasis, hepatic damage, and cetuximab [24], this reduction aligns with changes caused by PVL. According to Iluz-Freundlich et al., adult patients with chronic liver disease or reduced liver function, as is the case in this study, show a decrease in ALP levels [56].

Regarding glucose, although cetuximab may influence tumor metabolism [57], particularly by reducing glucose uptake in sensitive tumor cells, no significant direct effects on glucose or lactate metabolism have been previously reported. In our experimental model, the hypoglycemia observed after PVL is more likely to reflect perioperative stress or the increased metabolic demands associated with liver regeneration, rather than a direct effect of cetuximab [57, 58]. Weinebren et al. stated that hepatic resection greater than 67% (partial hepatectomy) causes mild hypoglycemia in rats, which becomes severe when resections exceed 82% (subtotal hepatectomy). Therefore, in our 50% PVL model, the effect would resemble that of a partial hepatectomy [59].

In processes like two-stage surgeries or partial hepatectomies, hepatic hypertrophy can promote the growth or reactivation of dormant micrometastases in the liver [60, 61]. Our group showed that while hepatic resection for macroscopic metastases improves short- and medium-term survival, it may also unintentionally stimulate the growth of dormant or undetected micrometastases due to the release of growth factors required for liver regeneration [16]. Similar findings were reported by Rigual et al. and Shi et al. [62, 63]. Most recurrences occur within 6 months post-surgery, supporting this theory, although their growth related to hepatic regeneration remains understudied. Based on our hypothesis, cetuximab could potentially prevent micrometastases reactivation and growth. Its use in the inter-surgical period during two-stage surgery might be beneficial without impairing liver regeneration. The therapeutic regimen using cetuximab as neoadjuvant therapy in CRLM is not currently described in the literature or clinical guidelines, so further studies are recommended to confirm its inter-surgical benefits and evaluate its potential in clinical trials [5].

Finally, some limitations warrant consideration. First, this study relied on a small-rodent PVL model, which—despite being widely used to investigate future liver remnant hypertrophy—differs from human anatomy and physiology in key aspects, including lobular organization, metabolic rate, and markedly accelerated regenerative kinetics. These species-specific characteristics may limit direct extrapolation to clinical liver regeneration after PVL. Second, unlike clinical regimens that employ repeated cycles of combination chemotherapy, cetuximab was administered as a single intra-arterial dose, which does not capture the systemic pharmacokinetics, cumulative effects, or potential toxicities associated with standard oncologic protocols. Third, the metabolic and stress responses to surgery in rodents may not mirror those in human liver surgery, given interspecies differences in glucose homeostasis and perioperative physiology. Nonetheless, the model offers relevant strengths, including controlled tumor implantation, reproducible PVL-induced hypertrophy, and the ability to isolate early interactions between regeneration and tumor biology. Together, these limitations underscore the need for further translational and clinical studies to confirm whether perioperative cetuximab can safely modulate tumor progression without impairing liver recovery in patients with CRLM [64–66].

## Conclusion

To conclude, in a murine model of selective PVL, neoadjuvant cetuximab did not impair hepatic mass recovery but modified hepatocyte nuclear DNA content. Administered seven days before PVL, it inhibited the proliferative stimulus on tumor implants triggered by regeneration-associated growth factors. The treatment did not significantly affect hepatic cytolysis or cholestasis markers, though it was associated with reduced glucose levels. Given the risk of micrometastases activation during liver regeneration, perioperative cetuximab may offer an oncological advantage in CRLM management.

## Data Availability

All data generated or analyzed during this study are included in this article. Further enquiries can be directed to the corresponding author.
